# Impact of de-escalation therapy on clinical outcomes for intensive care unit-acquired pneumonia

**DOI:** 10.1186/cc10072

**Published:** 2011-03-02

**Authors:** Mi Kyong Joung, Jeong-a Lee, Soo-youn Moon, Hae Suk Cheong, Eun-Jeong Joo, Young-Eun Ha, Kyung Mok Sohn, Seung Min Chung, Gee Young Suh, Doo Ryeon Chung, Jae-Hoon Song, Kyong Ran Peck

**Affiliations:** 1 Division of Infectious Diseases, Samsung Medical Center, Sungkyunkwan University School of Medicine, 50 Ilwon-dong, Gangnam-gu, Seoul 135-710, Republic of Korea; 2 Division of Infectious Diseases, Konyang University Hospital, 685 Gasuwon-dong, Seo-gu, Daejeon 302-718, Republic of Korea; 3 Division of Infectious Diseases, Kyunghee University Hospital, Hoegi-dong, Dongdaemun-gu, Seoul 130-702, Republic of Korea; 4 Division of Infectious Diseases, Konkuk University Hospital, 4-12 Hwawang-dong, Gangjin-gu, Seoul 143-701, Republic of Korea; 5 Division of Infectious Diseases, Chungnam National University Hospital, 33 Munhwa-ro, Jung-gu, Daejeon 301-721, Republic of Korea; 6 Divisions of Pulmonary and Critical Care Medicine, Samsung Medical Center, Sungkyunkwan University School of Medicine, 50 Ilwon-dong, Gangnam-gu, Seoul 135-710, Republic of Korea; 7 Asian-Pacific Research Foundation for Infectious Diseases, 50 Ilwon-dong, Gangnam-gu, Seoul 135-710, Republic of Korea

## Abstract

**Introduction:**

De-escalation therapy is a strategy currently used for the management of nosocomial pneumonia. In this study, we evaluated clinical outcomes and risk factors related to de-escalation therapy in patients with intensive care unit (ICU)-acquired pneumonia.

**Methods:**

This was a retrospective observational cohort study of ICU patients who developed pneumonia more than 48 hours after admission to the ICU at Samsung Medical Center from September 2004 to December 2007.

**Results:**

The 137 patients comprised 44 (32.1%) who received de-escalation therapy and 93 in the non-de-escalation group. The de-escalation group showed a lower pneumonia-related mortality rate than the non-de-escalation group by day 14 (2.3% vs. 10.8%, respectively; *P *= 0.08) and by day 30 (2.3% vs. 14%, respectively; *P *= 0.03) after the diagnosis of pneumonia. The variables independently associated with ICU-acquired pneumonia-related mortality included the Acute Physiology and Chronic Health Evaluation II (APACHE II) score and the modified Clinical Pulmonary Infection Score (CPIS) after 5 days with pneumonia. The non-de-escalation group had significantly higher APACHE II score and modified CPIS after 5 days with ICU-acquired pneumonia compared to the de-escalation group. Among all patients, 20.4% (28 of 137) had negative cultures for pathogens, and 42.9% (12 of 28) received de-escalation therapy. The latter 12 patients received de-escalation therapy and survived 30 days after the diagnosis of pneumonia.

**Conclusions:**

Patients in the de-escalation group showed a significantly lower mortality rate compared to patients in the non-de-escalation group. De-escalation therapy can be safely provided to patients with ICU-acquired pneumonia if they are clinically stable by day 5, even in those whose respiratory specimen cultures yield no specific pathogens.

## Introduction

Nosocomial pneumonia accounts for almost one-half of all intensive care unit (ICU) mortality and approximately 60% of mortality due to all nosocomial infections. The initial choice of antimicrobial therapy is critical to the clinical outcome of patients with nosocomial pneumonia. Early and aggressive empirical therapy with broad-spectrum agents targeted at the likely pathogens has been associated with a reduction in the ventilator-associated pneumonia (VAP) mortality rate [1-9]. Awareness of the need for early and appropriate therapy, however, may tempt the clinician to use aggressive empirical therapy at the first sign of infection. Such empirical practices could create a vicious cycle of early and aggressive broad-spectrum antibiotic therapy that may in turn lead to overuse of antibiotics and an increase in antimicrobial resistance.

De-escalation therapy is a method currently used for the management of serious infections, especially in nosocomial pneumonias [2,10-17]. Early administration of broad-spectrum antibiotics has been used for treatment to improve appropriate use of empirical therapy. Sequential de-escalation provides maximum benefit for the individual patient and reduces the selection pressure fueling the development of resistance. De-escalation strategies provide clinical balance between using broad-spectrum empirical antimicrobial agents and delaying the initiation of targeted therapy pending the bacteriological culture results. Several studies have shown that de-escalation therapy leads to reduced antibiotic use, shorter duration of therapy and reduced mortality [12,13,17].

The most recent VAP treatment guidelines of the American Thoracic Society (ATS) and the Infectious Diseases Society of America (IDSA) include recommendations for early, appropriate, broad-spectrum coverage and subsequent de-escalation of antibiotic regimens when possible, based on microbiological culture findings [18].

ATS and IDSA VAP treatment guidelines have suggested that negative lower respiratory tract cultures can be used to discontinue antibiotic therapy in a patient who shows clinical improvement at 48 to 72 hours after the diagnosis of pneumonia and has cultures obtained, in the absence of antibiotic initiation or change, over the previous 72 hours [12,15,18]. However, the outcome of de-escalation therapy in patients with negative cultures has not previously been reported.

Studies of de-escalated antimicrobial therapy based on antimicrobial sensitivity testing of microbiological cultures have reported that de-escalation therapy is not possible in patients with negative cultures [14]; that is, the outcome of patients with ICU-acquired pneumonia with negative cultures is unfavorable.

In this study, we evaluated pneumonia-related mortality at day 14 after diagnosis of pneumonia and the risk factors associated with pneumonia-related mortality among patients with ICU-acquired pneumonia that were managed with de-escalation therapy. Furthermore, we focused on the outcome of de-escalation therapy in patients with ICU-acquired pneumonia who had negative cultures.

## Materials and methods

### Study design and patients

This retrospective observational cohort study was conducted in 30-bed medical and surgical ICUs at Samsung Medical Center, a tertiary care university hospital, from September 2004 to December 2007. Patients were enrolled in the study if they were at least 18 years of age and the physicians established a diagnosis of ICU-acquired pneumonia that occurred more than 48 hours after admission to the ICU that required antibiotic treatment. Data collected included patient demographics, underlying disease, hospital and ICU admission dates, diagnosis at the time of ICU admission, chest radiographic findings, microbiological cultures, antimicrobial therapy prior to and during the ICU stay, duration of mechanical ventilation prior to and after the diagnosis of pneumonia and severity-of-illness indices, including the Acute Physiology and Chronic Health Evaluation II (APACHE II) score [19], the modified clinical pulmonary infection score (CPIS) [20] and the Charlson comorbidity index score (CCS) [21]. The baseline CPIS and APACHE II scores on day 5 after the diagnosis of pneumonia (5-day CPIS and 5-day APACHE II scores) were calculated. The APACHE II score was classified as category 1, ≤19; category 2, 20 to 23; and category 3, ≥24. The CPIS score was classified as category 1, ≤6; category 2, 7 to 9; and category 3, ≥10. Ethical approval for the study was granted by the Samsung Medical Center. The need for informed consent was waived because the study required no intervention and no breach of privacy or anonymity.

### Definitions

ICU-acquired pneumonia was diagnosed on the basis of new pulmonary infiltrates plus at least two of the following criteria: fever ≥38°C, blood leukocytes ≥10,000/mm^3 ^or ≤3,000/mm^3 ^and purulent tracheal secretions occurring more than 48 hours after admission to the ICU and within 72 hours of discharge from the ICU [22]. Only patients with first episodes of ICU-acquired pneumonia were eligible for the study. Appropriate antibiotic coverage was considered when at least one effective drug was included in the antibiotic treatment. Pneumonia-related deaths were considered related to the pulmonary infection if they occurred before any objective response to the antimicrobial therapy or if the pulmonary infection was considered a contributing factor to death in patients with comorbidity [3]. Each death summary was independently reviewed by two study investigators who were blinded to the use of de-escalation treatment. Mortality was classified as pneumonia-related if pneumonia was an immediate or underlying cause of death or if it played a major role in the patient's death. Mortality was defined as pneumonia-unrelated if the pneumonia was neither an immediate nor an underlying cause of death and played only a minor role, no role or an unknown role in the cause of death [23]. The overall mortality included all deaths that occurred during hospitalization.

### De-escalation therapy

De-escalation was defined as streamlined antibiotic treatment driven by microbiological documentation, clinical data and the severity-of-illness index achieved by decreasing the number and/or spectrum of antibiotics. This approach to the management of ICU-acquired pneumonia involves changing the focus from the use of multiple agents to the use of a single agent if *Pseudomonas aeruginosa *is not present, shortening the therapy to <5 days if the culture is negative and there have been >48 hours of defervescence, and changing from a broad to a narrow agent in the light of culture data [24]. Accordingly, patients receiving carbapenem were de-escalated to piperacillin and tazobactam, and patients receiving piperacillin and tazobactam were de-escalated to cefepime or a third-generation cephalosporin. Patients receiving combination therapy were de-escalated and switched to monotherapy by withholding fluoroquinolone, aminoglycosides or glycopeptides [14].

### Microbiological data collection

Microbiological data for the patients was obtained from cultures of transendotracheal aspirates (TAs), blood, pleural fluids and bronchoalveolar lavage (BAL) fluids. A bacteriological diagnosis required one or more of the following criteria: TA cultures with ≥10^5 ^colony-forming units (CFU)/ml, BAL cultures with ≥10^4 ^CFU/ml, blood or pleural fluid cultures with the same pathogen as the respiratory samples, histopathological evidence of pneumonia, positive urinary antigens of *Streptococcus pneumonia *or *Legionella pneumophila *and, for eligible specimens of TA, a white blood cell count WBC >25 and <10 epithelial cells per low-power field.

### Outcome criteria

The primary outcome measure was pneumonia-related mortality at day 14 after the diagnosis of pneumonia. The secondary outcomes included overall mortality and length of mechanical ventilator support. At the time of the diagnosis of pneumonia and the initiation of de-escalation therapy, the severity of illness was calculated using the APACHE II score and CPIS.

### Statistical analyses

Continuous variables were compared using Student's *t*-test for normally distributed variables and the Wilcoxon rank-sum test for non-normally distributed variables. The χ^2 ^statistic or Fisher's exact test was used to compare categorical variables. Multivariate analysis was performed using Cox regression analysis. The results of the statistical analysis are reported as adjusted hazard ratios (HRs) with 95% confidence intervals (95% CIs). All *P *values ≤0.05 were considered to indicate statistical significance. PASW for Windows software package version 17.0 (SPSS, Chicago, IL, USA) was used for the analyses.

## Results

One hundred thirty-seven patients (mean age, 61.0 ± 16.1 years) were included in the study. Ninety-seven (70.8%) of the patients were male. The patients had a median APACHE II score and modified CPIS of 15.0 ± 5.4 and 8.0 ± 1.5, respectively, at the time of the diagnosis of pneumonia. The median duration of mechanical ventilation and ICU stay before the diagnosis of pneumonia was 6.0 ± 8.9 days and 9.0 ± 20.9 days, respectively. The most frequent ICU admission diagnoses included general postoperative care (35.8%), neurological diseases (24.8%), cardiac diseases (13.1%) and non-ICU-acquired pneumonia (13.1%).

De-escalation therapy was administered in 44 patients (32.1%). Basic demographic and clinical characteristics of the de-escalation and non-de-escalation groups are summarized in Table 1. There were no differences in terms of prior length of ICU stay, prior antibiotic use, use of mechanical ventilation, onset of pneumonia, CCS, APACHE II score, modified CPIS and demographic characteristics between the two groups at the time of diagnosis of ICU-acquired pneumonia.

**Table 1:** Clinical characteristics of the de-escalation group and the non-de-escalation group in patients with ICU-acquired pneumonia^a^

Characteristics	De-escalation group (*N *= 44)	Non-de-escalation group (*N *= 93)	*P *value
Mean age (±SD), yr	57.45 ± 17.5	59.02 ± 15.4	0.45
Male:female ratio, *n*	30:14	67:26	0.64
Underlying conditions, *n *(%)			
Diabetes mellitus	8 (18.2%)	14 (15.0%)	
Structural lung disease	4 (9.1%)	9 (9.7%)	
Renal failure (Cr >2.0 mg/dl)	4 (9.1%)	19 (20.4%)	
Malignancy	10 (22.7%)	34 (36.6%)	
Liver disease	4 (9.1%)	8 (8.6%)	
Transplantation	5 (11.4%)	8 (8.6%)	
Congestive heart failure	10 (22.7%)	15 (16.1%)	
Postoperative state	24 (54.5%)	59 (63.4%)	
Cerebrovascular accident	12 (27.3%)	22 (23.7%)	
Alcoholism	1 (2.3%)	3 (3.2%)	
Prior antibiotic use, *n *(%)	42 (95.9%)	86 (92.5%)	0.51
Mean prior length of ICU stay (±SD), days	13.43 ± 22.7	13.39 ± 20.0	0.88
Use of MV, *n *(%)	39 (88.6%)	85 (91.4%)	0.60
Mean prior length of MV (±SD), days	7.6 ± 6.4	7.9 ± 9.8	0.37
Mean CCS (±SD)	2.43 ± 1.5	2.34 ± 1.5	0.66
Mean APACHE II score (±SD)	15.6 ± 5.5	15.3 ± 5.3	0.90
Mean CPIS (±SD)	8.32 ± 1.6	8.46 ± 1.3	0.06

^a^APACHE II, Acute Physiology and Chronic Health Evaluation II score; CCS, Charlson comorbidity index score; CPIS, Clinical Pulmonary Infection Score; Cr, creatinine; ICU, intensive care unit; MV, mechanical ventilation; SD, standard deviation.

Appropriate initial antibiotics were received by 32 patients (72.7%) in the de-escalation group and 63 patients (67.7%) in the non-de-escalation group (*P *= 0.55). Twelve patients in the de-escalation group received initial inappropriate therapy. Three patients did not receive anti-methicillin-resistant *Staphylococcus aureus *(anti-MRSA) antimicrobial therapy after the identification of MRSA. The therapy of three other patients was altered to include trimethoprim and sulfomethoxazole (TMP/SMX) because they had *Sternotrophomonas maltophilia*. Two patients showed resistance to the initial antibiotics, and four patients presented with carbapenem-resistant Gram-negative bacilli.

The rate of nonpneumonia infection during treatment of ICU-acquired pneumonia was 31.8% (14 of 44 patients) in the de-escalation group and 23.7% (22 of 93 patients) in the non-de-escalation group (*P *= 0.31). The median timing of de-escalation was 5.5 days. The mean modified 5-day CPIS and 5-day APACHE II score for all patients are summarized in Table 2. The mean changes in CPIS (from the day of diagnosis of pneumonia to day 5 after the diagnosis of pneumonia) in the de-escalation and non-de-escalation groups were 1.8 ± 1.8 and 1.2 ± 1.6, respectively (*P *= 0.7).

**Table 2:** Comparison of the APACHE II score and the modified CPIS at day 5 of pneumonia diagnosis between the two groups^a^

Severity index	De-escalation	Non-de-escalation	*P *value
Mean APACHE II score (±SD)	13.6 ± 4.4	15.8 ± 6.0	0.03
APACHE II score, *n *(%)			0.04
<19	34 (87.2%)	55 (72.4%)	
19 to 23	4 (10.3%)	11(14.5%)	
>23	1 (2.6%)	10 (13.2%)	
Mean CPIS (±SD)	6.5 ± 1.2	7.5 ± 1.4	0.002
CPIS category, *n *(%)			0.009
4 to 6	21 (48.8%)	25 (27.8%)	
7 to 9	22 (51.2%)	61 (67.8%)	
≥10	0 (0%)	4 (2.7%)	

^a^APACHE II, Acute Physiology and Chronic Health Evaluation II; CPIS, Clinical Pulmonary Infection Score.

There were 26 patients (19%) with early ICU-acquired pneumonia (≤4 days following ICU admission) and 111 patients (81%) with late ICU-acquired pneumonia (≥5 days following ICU admission). De-escalation was performed in 9 (34.6%) of 26 patients with early ICU-acquired pneumonia and in 35 (31.5%) of 111 patients with late ICU-acquired pneumonia (*P *= 0.76). The rate of de-escalation was 36.5% (27 of 74) of the episodes in the medical ICU and 27.0% (17 of 63) in the surgical ICU (*P *= 0.273).

Of the 137 patients, 117 microbiological pathogens were isolated from 109 patients (79.6%). The initial microbiological identification was based on quantitative information from the TA in 99 cases (73.8%), BAL fluid samples in 5 cases (3.6%), and blood cultures in 3 cases (2.2%). MRSA was the most common pathogen identified (*n *= 44, 37.6%), followed by *Acinetobacter *spp. (16.2%), *Pseudomonas *spp. (15.4%), *Klebsiella pneumoniae *(10.3%) and *S. maltophilia *(6.8%).

De-escalation was performed in 32 (29.4%) of 109 patients with positive cultures and in 12 (42.9%) of 28 patients with negative cultures. De-escalation was initiated in 34.5% of episodes with potentially resistant pathogens (nonfermenting Gram-negative bacilli (NFGNB) and MRSA) compared to 65.5% among the remaining pathogens (*P *= 0.45). Among the 95 patients in whom the antibiotics could be de-escalated, based on the antimicrobial susceptibility data, 32 patients (33.7%) received de-escalation therapy.

The most frequently prescribed empirical antibiotic was carbapenem (35%), followed by piperacillin and tazobactam (28.5%), third-generation cephalosporin (22.6%) and cefepime (6.6%). Vancomycin was prescribed in 60 cases (43.8%) as part of a combination regimen. The proportion of cases with appropriate initial antibiotic treatment was 69.3% (95 of 137). A two-agent combination regimen was prescribed in 103 (75.2%) of the total group of patients: 37 in the de-escalation group and 66 in the non-de-escalation group. Monotherapy was prescribed in 32 (23.4%) of all patients: 7 in the de-escalation group and 25 in the non-de-escalation group. Two patients received three antibiotics as empirical therapy. There was no difference between the groups with regard to the use of combination therapy versus monotherapy.

De-escalation therapy in 44 patients was implemented by decreasing the number of antibiotics, the spectrum of antibiotics or both. The number of antibiotics used was decreased for 25 patients (56.8%). In the majority of cases, vancomycin was discontinued when MRSA was not identified. Antibiotics were streamlined to a narrow spectrum in 12 patients (27.3%), whereas 7 patients (15.9%) received de-escalation therapy by decreasing the number as well as the spectrum of antibiotics. Thirty-four of the 137 patients died during a 30-day follow-up period. This represented an overall mortality rate of 24.8% (34 of 137) and a pneumonia-related mortality rate of 10.2% (14 of 137).

**Figure 1:**
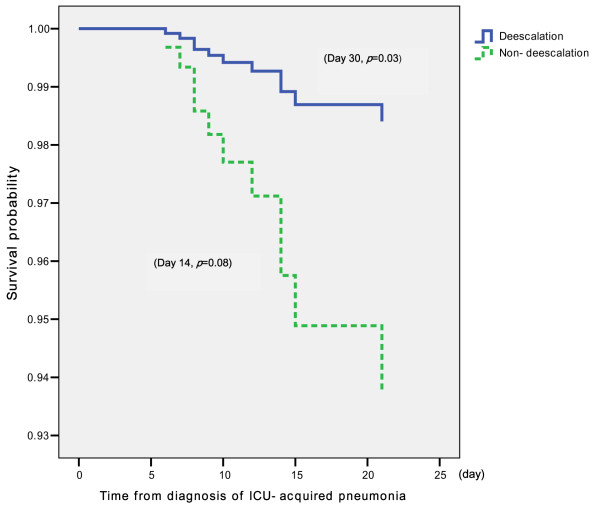
**Pneumonia-related mortality between the de-escalation group and the non-de-escalation groups**.

Although there was a lower trend for the pneumonia-related mortality rate in the de-escalation group, the difference did not reach statistical significance by day 14 after the diagnosis of ICU-acquired pneumonia (1 (2.3%) of 44 patients versus 10 (10.8%) of 93 patients; *P *= 0.08) (Figure 1). The pneumonia-related mortality at day 30 was significantly lower in the de-escalation group than in the non-de-escalation group (1 (2.3%) of 44 patients versus 13 (14%) of 93 patients; *P *= 0.03) (Figure 1). With regard to overall mortality, the de-escalation group had a significantly lower mortality rate than the non-de-escalation group by day 14 (*P *= 0.04) and by day 30 (*P *= 0.01).

The pneumonia-related mortality was associated with inadequate empirical antibiotics, non-de-escalation of antibiotics, baseline APACHE II score, 5-day APACHE II score and 5-day CPIS on the basis of univariate analysis. However, only the 5-day APACH E II score and the 5-day CPIS were found to be independent risk factors associated with pneumonia-related mortality and overall mortality (Table 3).

**Table 3:** Factors associated with 30-day pneumonia-related mortality in patients with ICU-acquired pneumonia determined by multivariable analysis^a^

Variable	Adjusted hazard ratio^b^	95% CI	*P *value
Inadequacy of antibiotics	2.145	0.483 to 9.536	0.316
Non-de-escalation of antibiotics	3.988	0.047 to 6.985	0.245
Baseline APACHE II score (reference score <19)			0.198
20 to 23	2.528	0.609 to 10.493	0.201
≥24	7.611	0.615 to 94.179	0.114
5-day APACHE II score (reference score <19)			0.011
20 to 23	4.934	0.974 to 25.003	0.054
≥24	12.839	2.359 to 69.883	0.003
5-day CPIS (reference score 4 to 6)			0.017
7 to 9	2.154	0.361 to 12.861	0.400
≥10	26.782	2.180 to 329.011	0.010

^a^ICU, intensive care unit; 95% CI, 95% confidence interval; APACHE II, Acute Physiology and Chronic Health Evaluation II; CPIS, Clinical Pulmonary Infection Score; ^b^Cox proportional hazard regression analysis was used to determine the relationship between mortality and independent baseline variables identified in univariable analysis, including inadequacy of initial antibiotics, de-escalation of antibiotics, baseline APACHE II score, 5-day APACHE II score and 5-day CPIS.

The APACHE II score and the modified CPIS in patients with negative cultures are outlined in Table 4. There was a downward trend in the scores of the patients in the de-escalation group; however, the difference was not statistically significant when compared to the non-de-escalation group. The 30-day pneumonia-related mortality of patients with negative cultures was 0 of 12 (0%) in the de-escalation group and 2 of 16 (12.5%) in the non-de-escalation group. Two patients with negative cultures died on day 3 and day 25, respectively, and they had relatively high APACHE II scores and modified CPIS on day 5.

**Table 4:** Comparison of severity-of-illness index in patients with negative cultures^a^

Severity index	De-escalation group (*N *= 12)	Non-de-escalation group (*N *= 16)	*P *value
Mean baseline APACHE II score (±SD)	15.36 ± 5.9	16.56 ± 6.6	0.63
Mean baseline CPIS (±SD)	6.83 ± 1.2	6.56 ± 0.9	0.50
5-day APACHE II score (±SD)	11.70 ± 5.0	13.82 ± 3.3	0.26
Category 1, n (%)	11 of 12 (91.7%)	14 of 16 (87.5%)	0.90
Category 2, n (%)	1 of 12 (8.3%)	2 of 16 (12.5%)	
Mean 5-day CPIS (±SD)	5.9 ± 1.1	6.5 ± 1.2	0.23
Category 1, n (%)	9 of 12 (75%)	8 of 16 (50%)	0.13
Category 2, n (%)	3 of 12 (25%)	8 of 16 (50%)	

^a^APACHE II, Acute Physiology and Chronic Health Evaluation II; CPIS, Clinical Pulmonary Infection Score.

## Discussion

In this study, the overall de-escalation rate was 32.1% (44 of 137 patients). This proportion increased to 33.7% (32 of 95 patients) when only episodes in which de-escalation was applicable based on antimicrobial susceptibility were considered. The de-escalation rate in this study was lower than the rates in previous studies, in which the de-escalation rates of patients with susceptible pathogens have been reported to be 38% to 51.9% [11,14]. Alvarenz-Lerma *et al. *[11] reported that a low de-escalation rate is probably due to a high prevalence of *Pseudomonas *spp. infection (30.6%), intensive prior use of antibiotics (79.1%) and a larger proportion of late-onset episodes (90.6%). Our study has shown a high incidence of NFGNB cases, including *Pseudomonas *spp. infection (38.4%) and MRSA infection (37.6%). In addition, a high rate of prior use of antibiotics (93.4%) and a larger proportion of cases with late ICU-acquired pneumonia (81%) were evident in our study. The low de-escalation rate in this study might be due to these factors.

Currently, de-escalation is significantly less frequent in patients with pneumonia who have NFGNB and MRSA. This is because the effectiveness of this approach varies according to local patterns of antibiotic sensitivity. Although Korea has a relatively high prevalence of multiresistant pathogens [25,26], the de-escalation rate in patients with NFGNB and MRSA in the present study was 34.5%, which is much higher than the 2.7% and 23.1% rates described in previous reports [11,14].

Among 44 patients in the de-escalation group, 12 patients received inappropriate initial antibiotics. Two patients were changed to narrow-spectrum antibiotics because they showed resistance to initial antibiotics. Four patients who harbored carbapenem-resistant, Gram-negative bacilli received carbapenem or piperacillin and tazobactam, because colistin was not available at that time. Two of these patients died.

Three patients did not receive anti-MRSA antimicrobial agents, even though MRSA infection was identified, because the patients were improving with initial antibiotics. All three patients survived. The MRSA isolated from quantitative tracheal aspirate might not be true pathogens. If the bacteria were merely colonizing pathogens, but not the cause of infection, then the initial antimicrobial therapy would be inappropriate. Quantitative TA allows the identification of pathogens in the great majority (90%) of cases [14]. The antibiotic regimen of three other patients with *S. maltophilia *was changed to include TMP/SMX and the broad-spectrum antibiotics were switched to narrow-spectrum antibiotics.

The pneumonia-related mortality rate was not significantly different in the de-escalation group compared to the non-de-escalation group at day 14 (*P *= 0.08). The pneumonia-related mortality and overall mortality at day 30, however, was significantly lower in the de-escalation group (*P *= 0.03). This finding is consistent with the results reported by Kollef *et al. *[13]. The pneumonia-related mortality was associated with the 5-day APACHE II score and the 5-day CPIS, even with the adjustment for other factors in addition to the scores at baseline in this study.

The 5-day APACHE II score and the 5-day CPIS were significantly lower in the de-escalation group compared to the non-de-escalation group. The cause of high mortality in the non-de-escalation group was probably related to the high APACHE II score and modified CPIS on day 5, as well as the timing of de-escalation (Table 2). This suggests that de-escalation is effective in patients with ICU-acquired pneumonia who have a more stable severity-of-illness index on days 3 to 5 after the diagnosis of pneumonia. The mean changes in CPIS in the de-escalation group were higher, although this result was statistically insignificant, than in the non-de-escalation group. De-escalation was not performed in patients with a category 3 APACHE II score and CPIS at the time of de-escalation. However, patients in category 1 can be safely de-escalated, and those in category 2 can be considered for de-escalation on the basis of the clinical response of the pneumonia.

De-escalation was carried out in 12 (42.9%) of 28 patients with negative cultures, which resulted in no mortality. Rello *et al. *[14] did not perform de-escalation in patients with negative cultures because of concern about false-negative cultures and the time to resolution of the febrile illness. Alvarenz-Lerma *et al. *[11] also did not perform de-escalation in 113 patients with negative cultures, which resulted in a prolonged administration of imipenem. The high portion of patients with negative cultures who did not receive de-escalation was probably influenced by the lack of specific recommendations for de-escalation.

Although our data were collected from a small number of patients with negative cultures, more than 40% of the patients received de-escalation therapy, and all 12 patients survived at day 30 after the diagnosis of pneumonia. The de-escalation group had slightly lower APACHE II score and a lower modified CPIS trend at the time of de-escalation compared to the non-de-escalation group, although both were not statistically significant. Among all patients with negative cultures, two patients in the non-de-escalation group died. They had category 3 APACHE II scores and modified CPIS. If patients with negative cultures are classified in category 1 of the APACHE II score and modified CPIS by day 5, the findings of this study suggest that de-escalation could be considered for such patients with ICU-acquired pneumonia. This is the first study to show that de-escalation therapy in patients with negative cultures is feasible in patients with stable APACHE II scores and modified CPIS.

This study has several limitations. First, because it was a retrospective cohort study, there was no control group. It is ethically impossible to randomize ICU patients because patients with ICU-acquired pneumonia are seriously ill and require complex care. These limitations were minimized by the multivariate analysis. Second, it is difficult to distinguish true infection from colonization. Our respiratory tract specimens were mainly TA (73.8%). It has been noted above that three MRSA isolates in the de-escalation group might not have been true pathogens. It is somewhat confusing to say that therapy was inappropriate because it is possible that MRSA was a colonizer. However, we performed microbiological identification using quantitative methods, and the number of BAL specimens from patients was similar to those described in previous reports. Rello *et al. *[14] reported that quantitative TA and bronchoscopic samples allowed identification of pathogens in 90% and 93% of episodes, respectively, and there were no differences between the patients diagnosed on the basis of quantitative TA or bronchoscopy with regard to crude mortality and ICU mortality data. Third, there are no standard criteria for initial empirical antibiotic treatments and the timing of de-escalation. However, clinicians usually attempt to follow the currently available ATS and IDSA VAP guidelines for the diagnosis and treatment of pneumonia. Fourth, because of the small number of patients and because it was a single-center study, the results might not be applicable to other groups. Fifth, we measured the modified clinical pulmonary infection score retrospectively. The scores of tracheal secretions were classified as 0 (rare), 1 (abundant) and 2 (abundant and purulent) using ICU nursing records [20]. The ICU nurses recorded the hourly full particles of patients' information. For example, tracheal secretions were recorded as scant whitish, large whitish or large yellowish secretions. We reviewed the nursing records and decided on a score of tracheal secretions.

## Conclusions

The patients in the de-escalation group did not show increased mortality compared to those in the non-de-escalation group. The results of this study suggest that de-escalation therapy based on the APACHE II score and the modified CPIS 5 days after the diagnosis of pneumonia can be safely applied with good clinical outcomes for patients with ICU-acquired pneumonia, even in those with negative cultures. Prospective, large studies are needed to evaluate the efficacy of the de-escalation strategy in patients with negative cultures.

## Key messages

• The initial choice of antimicrobial therapy is critical to the clinical outcomes of patients with nosocomial pneumonia.

• Awareness of the need for early and appropriate therapy may tempt the clinician to use aggressive empirical therapy at the first sign of infection.

• De-escalation therapy is a method currently used for the management of serious infections, especially in patients with nosocomial pneumonia.

• De-escalation therapy based on APACHE II score and the modified CPIS 5 days after the diagnosis of pneumonia can be safely applied with good clinical outcomes among patients with ICU-acquired pneumonia, even in those patients with negative cultures.

• Prospective, large studies are needed to evaluate the efficacy of the de-escalation strategy in patients with ICU-acquired pneumonia.

## Abbreviations

APACHE II: Acute Physiology and Chronic Health Evaluation II; ATS: American Thoracic Society; CCS: Charlson comorbidity index score; CI: confidence interval; CPIS: Clinical Pulmonary Infection Score; HR: hazard ratio; IDSA: Infectious Diseases Society of America; MRSA: methicillin-resistant *Staphylococcus aureus*; MV: mechanical ventilation; NFGNB: nonfermenting Gram-negative bacilli; VAP: ventilator-associated pneumonia.

## Competing interests

The authors declare that they have no competing interests.

## Authors' contributions

MKJ, GYS and KRP contributed to study conception and design. MKJ, JL, HSC, SYM, EJJ, YEH, KMS and SMC collected and analyzed the data. MKJ drafted the manuscript. MKJ, SMC, JHS and KRP participated in the drafting and revision of the manuscript. All authors were involved in data acquisition and read and approved the final manuscript.

## References

[B1] Alvarez-Lerma F Modification of empiric antibiotic treatment in patients with pneumonia acquired in the intensive care unit. ICU-Acquired Pneumonia Study Group Intensive Care Med 1996 22 387 394 10.1007/BF01712153 8796388

[B2] Luna CM Vujacich P Niederman MS Vay C Gherardi C Matera J Jolly EC Impact of BAL data on the therapy and outcome of ventilator-associated pneumonia Chest 1997 111 676 685 10.1378/chest.111.3.676 9118708

[B3] Rello J Gallego M Mariscal D Soñora R Valles J The value of routine microbial investigation in ventilator-associated pneumonia Am J Respir Crit Care Med 1997 156 196 200 9230747 10.1164/ajrccm.156.1.9607030

[B4] Celis R Torres A Gatell JM Almela M Rodríguez-Roisin R Agustí-Vidal A Nosocomial pneumonia: a multivariate analysis of risk and prognosis Chest 1988 93 318 324 10.1378/chest.93.2.318 3338299

[B5] Ibrahim EH Sherman G Ward S Fraser VJ Kollef MH The influence of inadequate antimicrobial treatment of bloodstream infections on patient outcomes in the ICU setting Chest 2000 118 146 155 10.1378/chest.118.1.146 10893372

[B6] Iregui M Ward S Sherman G Fraser VJ Kollef MH Clinical importance of delays in the initiation of appropriate antibiotic treatment for ventilator-associated pneumonia Chest 2002 122 262 268 10.1378/chest.122.1.262 12114368

[B7] Kollef MH Sherman G Ward S Fraser VJ Inadequate antimicrobial treatment of infections: a risk factor for hospital mortality among critically ill patients Chest 1999 115 462 474 10.1378/chest.115.2.462 10027448

[B8] Leroy O Meybeck A d'Escrivan T Devos P Kipnis E Georges H Impact of adequacy of initial antimicrobial therapy on the prognosis of patients with ventilator-associated pneumonia Intensive Care Med 2003 29 2170 2173 10.1007/s00134-003-1990-x 13680112

[B9] Torres A Aznar R Gatell JM Jiménez P González J Ferrer A Celis R Rodriguez-Roisin R Incidence, risk, and prognosis factors of nosocomial pneumonia in mechanically ventilated patients Am Rev Respir Dis 1990 142 523 528 2202245 10.1164/ajrccm/142.3.523

[B10] Hoffken G Niederman MS Nosocomial pneumonia: the importance of a de-escalating strategy for antibiotic treatment of pneumonia in the ICU Chest 2002 122 2183 2196 10.1378/chest.122.6.2183 12475862

[B11] Alvarez-Lerma F Alvarez B Luque P Ruiz F Dominguez-Roldan JM Quintana E Sanz-Rodriguez C ADANN Study Group Empiric broad-spectrum antibiotic therapy of nosocomial pneumonia in the intensive care unit: a prospective observational study Crit Care 2006 10 R78 10.1186/cc4919 16704742 PMC1550932

[B12] Kollef MH Kollef KE Antibiotic utilization and outcomes for patients with clinically suspected ventilator-associated pneumonia and negative quantitative BAL culture results Chest 2005 128 2706 2713 10.1378/chest.128.4.2706 16236946

[B13] Kollef MH Morrow LE Niederman MS Leeper KV Anzueto A Benz-Scott L Rodino FJ Clinical characteristics and treatment patterns among patients with ventilator-associated pneumonia Chest 2006 129 1210 1218 10.1378/chest.129.5.1210 16685011

[B14] Rello J Vidaur L Sandiumenge A Rodríguez A Gualis B Boque C Diaz E De-escalation therapy in ventilator-associated pneumonia Crit Care Med 2004 32 2183 2190 15640629 10.1097/01.ccm.0000145997.10438.28

[B15] Singh N Rogers P Atwood CW Wagener MM Yu VL Short-course empiric antibiotic therapy for patients with pulmonary infiltrates in the intensive care unit: a proposed solution for indiscriminate antibiotic prescription Am J Respir Crit Care Med 2000 162 505 511 10934078 10.1164/ajrccm.162.2.9909095

[B16] Micek ST Ward S Fraser VJ Kollef MH A randomized controlled trial of an antibiotic discontinuation policy for clinically suspected ventilator-associated pneumonia Chest 2004 125 1791 1799 10.1378/chest.125.5.1791 15136392

[B17] Soo Hoo GW Wen YE Nguyen TV Goetz MB Impact of clinical guidelines in the management of severe hospital-acquired pneumonia Chest 2005 128 2778 2787 10.1378/chest.128.4.2778 16236955

[B18] American Thoracic Society; Infectious Diseases Society of America Guidelines for the management of adults with hospital-acquired, ventilator-associated, and healthcare-associated pneumonia Am J Respir Crit Care Med 2005 171 388 416 10.1164/rccm.200405-644ST 15699079

[B19] Knaus WA Draper EA Wagner DP Zimmerman JE APACHE II: a severity of disease classification system Crit Care Med 1985 13 818 829 10.1097/00003246-198510000-00009 3928249

[B20] Fartoukh M Maitre B Honoré S Cerf C Zahar JR Brun-Buisson C Diagnosing pneumonia during mechanical ventilation: the clinical pulmonary infection score revisited Am J Respir Crit Care Med 2003 168 173 179 10.1164/rccm.200212-1449OC 12738607

[B21] Charlson ME Pompei P Ales KL MacKenzie CR A new method of classifying prognostic comorbidity in longitudinal studies: development and validation J Chronic Dis 1987 40 373 383 10.1016/0021-9681(87)90171-8 3558716

[B22] Garner JS Jarvis WR Emori TG Horan TC Hughes JM CDC definitions for nosocomial infections, 1988 Am J Infect Control 1988 16 128 140 10.1016/0196-6553(88)90053-3 2841893

[B23] World Health Organization Manual of the International Statistical Classification of Diseases, Injuries, and Causes of Death 1977 Geneva, Switzerland: World Health Organization

[B24] Vidaur L Sirgo G Rodríguez AH Rello J Clinical approach to the patient with suspected ventilator-associated pneumonia Respir Care 2005 50 965 974 15972116

[B25] Lee K Lim CH Cho JH Lee WG Uh Y Kim HJ Yong D Chong Y High prevalence of ceftazidime-resistant *Klebsiella pneumoniae *and increase of imipenem-resistant *Pseudomonas aeruginosa *and *Acinetobacter *spp. in Korea: a KONSAR program in 2004 Yonsei Med J 2006 47 634 645 10.3349/ymj.2006.47.5.634 17066507 PMC2687749

[B26] Chong Y Lee K Present situation of antimicrobial resistance in Korea J Infect Chemother 2000 6 189 195 10.1007/s101560070001 11810564

